# Von Willebrand disease: A century of progress

**DOI:** 10.1016/j.rpth.2026.106790

**Published:** 2026-06-12

**Authors:** Caterina Casari, Brooke Sadler, Sophie Susen, Riitta Lassila

**Affiliations:** 1 Hémostase Inflammation Thrombose (HITh) U1176, INSERM, Université Paris-Saclay, Le Kremlin-Bicêtre, France; 2 Department of Pediatrics, Division of Hematology/Oncology, Washington University School of Medicine, St Louis, Missouri, USA; 3 Department of Hemostasis and Transfusion, Centre Hospitalier Universitaire Lille, Institut Pasteur de Lille, INSERM, University of Lille, Lille, France; 4 Research Program in Systems Oncology, Faculty of Medicine, Department of Hematology, Helsinki University Hospital, University of Helsinki, Helsinki, Finland

**Keywords:** bleeding, genetics, treatment, von Willebrand disease, von Willebrand factor

## Abstract

One hundred years after the initial description of von Willebrand disease, originally referred to as pseudohemophilia, this article is a tribute to Dr Erik von Willebrand and a testament to the progress in our understanding of von Willebrand factor. Main focuses have been on structure and hemostatic function, as well as the advancements in the diagnosis, genetics, and management of von Willebrand disease. Insightful early observations led to the discovery of the main VWF ligands and interaction domains and the molecular mechanisms controlling these associations in the context of hemostasis. Reflecting these intricate mechanisms, the genetics and diagnosis of von Willebrand disease remain challenging, especially for the mild, quantitative deficiencies. Treatment developments and innovations have historically progressed quite slowly and in the shadow of hemophilia. However, recent patient-centered studies underscoring unmet clinical needs have catalyzed a dynamic and rapidly evolving effort to improve patient care and clinical outcomes.

## Essentials

• Early observations made by Dr Erik von Willebrand 100 years ago remain relevant even today.
• Hemostatic functions of VWF have been described; however, novel ligands and multifactorial pathophysiological mechanisms continue to be discovered.
• Genetic studies and diagnosis remain challenging.
• To address unmet clinical needs, innovative studies and therapeutic developments aimed at improving patient care are emerging.

## Visual Abstract

**Figure:**
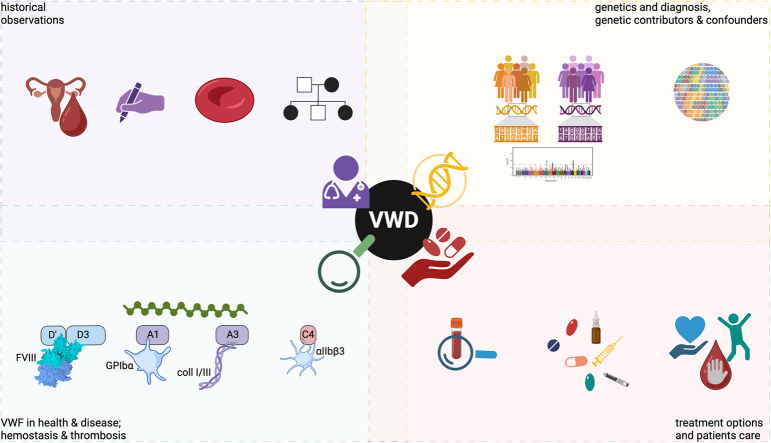

## 1 Reflecting on the Past

In February 1926, the Swedish-language Finnish journal “Finska Läkaresällskapets Handlingar” published the seminal original paper on pseudohemophilia, which was authored by Erik Adolf von Willebrand. This journal is still active, and it is a historical treasure to have the original publication in one’s bookshelf. Dr von Willebrand was an internist with a broad interest in medicine. He published 40 articles on hematology, blood cells and hemostatic defects, endocrinology, and beyond during 1899-1945. As one example on his medical entrepreneurship, he administered the first insulin injection for a diabetic patient in Finland. Curiously, von Willebrand also developed general rehabilitation methods—physiotherapy being important in bleeding disorders affecting joints and functional ability to prevent and manage the long-term consequences of bleeding complications.

**Figure 1:**
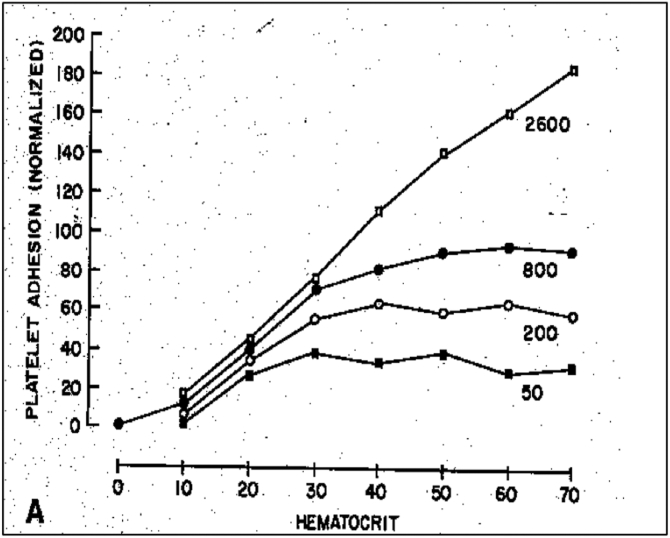
Role of red blood cells in supporting VWF interaction with platelets. Hematocrit above 30% provides increasing support for platelet interaction at vascular injury sites. If the VWF level is low and/or VWF-mediated platelet deposition is dysfunctional, low hematocrit worsens the hemostatic defect. Figure reproduced, with permission, from Turritto and Weiss [3]. VWF, von Willebrand factor.

In the original article about the “novel bleeding disorder” published 100 years ago, Erik von Willebrand drafted the famous family tree of von Willebrand disease (VWD)–affected family, already highlighting the hereditary traits of the disease. Moreover, the translational aspects in his original contribution are remarkable [1,2]. Namely, he very carefully dissected the hematopathological topics, including the mechanisms of hemostasis and the role of anemia, with the use of a blood flow assay (Figure 1, Table 1). Low numbers of red blood cells impair shear forces and contribute to the bleeding tendency with a concomitant defect or lack of VWF [3]. This is still a highly valuable concept to be considered, specifically in patients with recurrent bleeding, such as menstruation and gastrointestinal bleeding (GIB). Today, we benefit from the availability of prophylactic replacement therapy, interventions, and the proactive management of bleeding and anemia (Figure 1). However, there still appears to be room for optimizing their use.

**Table 1:** Eleven comprehensive diagnostic examinations by Erik von Willebrand interpreted for today.

In 1926	In 2026
1. Red and white blood cell count and differentiation, determination of hemoglobin	1. Hematology, exclusion of anemia and infection
2. Platelet count and morphology	2. Dg for thrombocytopenia and platelet size, peripheral blood smear
3. Platelet agglutination	3. Platelet agglutination (fixed platelets) or aggregation (PRP) induced by ristocetin (RIPA), VWF:IbM
4. Bleeding time (Duke method)	4. PFA200, platelet function analysis implementing whole blood and flow
5. Coagulation times	5. APTT, TT, and PT to exclude factor deficiencies
6. Clot retraction	6. Clot retraction
7. Capillary thrombometer, thrombosis time	7. T-TAS or other microfluidic studies
8. Subjection to stasis, Rumpel Leede experiment	8. Fibrinolytic capacity
9. Capillary microscopy	9. Capillary microscopy to assess vascular abnormalities and hairpin vessels
10. Plasma proteins and their distribution	10. VWF antigen and activity and fibrinogen, FXIII, and others according to APTT/PT screen
11. Analysis of blood group	11. Blood group 0 with low VWF adds to vulnerability of bleeds

Modified from von Willebrand EA [1].

APTT, activated partial thromboplastin time; PT, prothrombin time; PRP, platelet-rich plasma; RIPA, ristocetin-induced platelet aggregation; T-TAS, total thrombus-formation analysis system; TT, thrombin time; VWF, von Willebrand factor.

The first index case was a 5-year-old Hjördis, who was brought, from the island of Föglö in the Åland archipelago, to the Helsinki Deaconess hospital, where Erik von Willebrand was the clinical and laboratory director [4]. The family sought help for her generalized bleeding tendency—mainly nose and ankle bleeds—and anemia. Similar cases of severe bleeding in the family, with lethal outcome, were reported to him, as visualized in the famous family tree. These clinical observations intrigued von Willebrand, and he designed hematological studies focusing on blood coagulation, which were executed in Föglö with the help of his local assistant. Nine years later, at the age of 14 years, Hjördis sadly passed away due to her fourth but fatal, unstoppable menstrual bleed. It is likely that severe recurrent bleeding was causing anemia. The early deaths in the family continued haunting [3].

VWD was named after Erik von Willebrand by Margareta Blombäck (Stockholm, Sweden) and Inga Marie Nilsson (Malmö, Sweden), the two Nordic pioneers of bleeding disorders. The title “pseudohemophilia” of the original article was understandable but unfortunate, as it shadowed VWD and the significance of this condition, which was recognized years later as the most common inherited bleeding disorder. VWD remained in the shadow of hemophilia for several decenniums. Only more recently, VWD has obtained increasing attention, including the possibility for regular prophylaxis, awareness and care of VWD-associated women’s sex-specific bleeding issues, and a more generalized recognition of the need for novel therapeutic tools. While women are mostly affected by this disease, severe GIB, joint bleeds, and other bleeds occur equally in both men and women.

The initial suspicion and recognition of VWD are critical. These include the clinical presentation and its scrutinized assessment of generalized mucocutaneous bleeding symptoms, familial trait, and diagnostic laboratory and genetic approaches to design management. Determining whether low or dysfunctional VWF is a risk factor or a disease may be complicated by a concomitant prothrombotic or antihemostatic profile. The diagnostic criteria are critically important and need expert training, specialized laboratory assays, and rigorous interpretation. Treatment decisions, including prophylaxis and emergency issues related to surgery, heavy menstrual and gastrointestinal bleeds, pregnancy, and delivery, are still important specific topics, which need optimization, regular follow-up, shared decision-making, and multidisciplinary care.

### 1.1 Learnings by the original article

The main observations of Erik von Willebrand reflected his innovative approach, which remains true in VWD (Table 1). He introduced a multifactorial role for the vascular wall, platelet function, red blood cells, hemorheology, blood group, and blood flow in addition to the deficiency of a factor in plasma, which he identified as responsible for prolonging bleeding time while clotting occurred quite normally [1]. Considering all these aspects contributes to the optimal management of VWD.

After this historical perspective, our article will discuss the main roles of VWF in health and disease in the context of hemostasis. From this perspective, genetic considerations, contributors, and confounders, important to tailor individualized management, will be considered. Finally, treatment choices and clinical applications, including unmet clinical needs and the latest achievements in therapy, will be introduced.

## 2 Roles of Von Willebrand Factor in Health and Disease

### 2.1 VWF structure-function relationship in hemostasis

**Figure 2:**
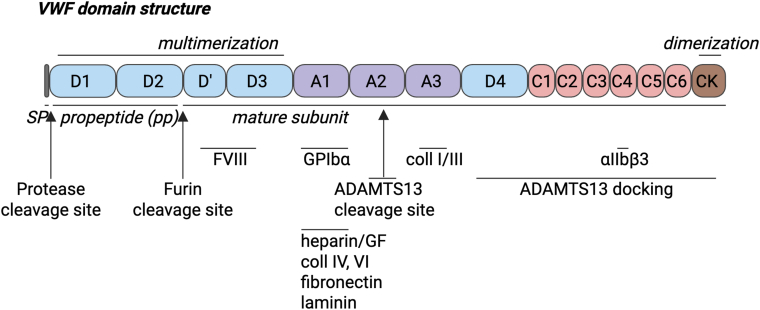
VWF domains and main ligands. Domains composing the VWF pre-pro-polypeptide according to Zhou et al. [103]. Cleavage sites responsible for the removal of the SP by an unidentified peptidase, Furin and ADAMTS13, are indicated by arrows. VWF domains containing binding sites for ligands mentioned in this article are also reported. Additional VWF ligands are reviewed in Atiq et al. [43]. Created in BioRender by C.C. (2026; https://BioRender.com/iv5ayw1). GF, heparin-binding growth factors; SP, signal peptide; VWF, von Willebrand factor.

VWF is a large adhesive glycoprotein with a unique structural-functional identity best known for its role in hemostasis. It is synthesized by endothelial cells and megakaryocytes as a pre-pro-polypeptide (Figure 2) and stored intracellularly or secreted as heterogeneous-sized multimers (>60 subunits) [5].

The multimeric nature of VWF has both physiologic and pathologic implications. The multimer size is directly associated with hemostatic functions, with large multimers being the most active and able to sustain interactions with major ligands. Consequently, the loss of high-molecular-weight multimers (HMWMs) is associated with bleeding, while inefficient multimer proteolysis is associated with the accumulation of ultralarge multimers and thrombotic complications. The large size and multimeric conformation of VWF are also essential for its mechanosensing properties [6]. An increase in local shear forces is necessary to switch VWF molecules from an inactive to a hemostatically active conformation, binding to platelet glycoprotein (GP)Ibα. However, the multimeric nature of VWF also makes it susceptible to genetic variants with dominant negative effects, which complexify genetic and clinical evaluations and disease classification [7].

**Figure 3:**
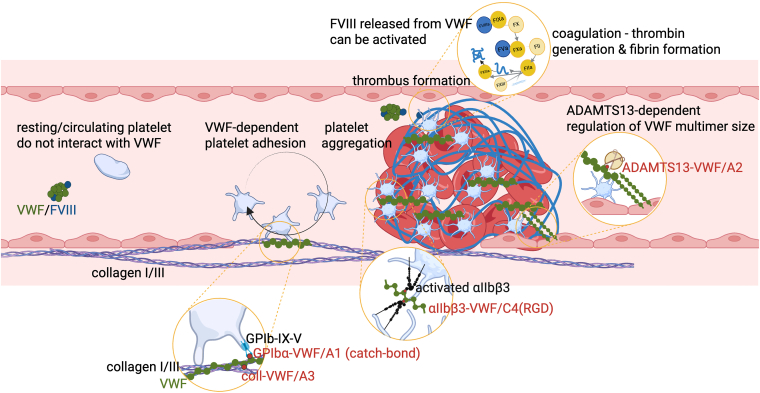
VWF interactions in hemostasis. Major VWF interactions important for the hemostatic process (discussed in this article) are illustrated as zoomed-in callouts. VWF interacts with many more ligands with implications in thrombosis, hemostasis, and beyond (reviewed in Atiq et at [43]). Created in BioRender by C.C. (2026; https://BioRender.com/l3mu3fu). VWF, von Willebrand factor.

In the physiologic state, VWF is present within specialized intracellular compartments (the Weibel-Palade bodies and the alpha granules in endothelial cells and platelets, respectively) in circulation and in the subendothelium, and its hemostatic functions are highly dependent on its ability to interact with various partners. Circulating, inactive VWF binds coagulation factor VIII (FVIII) with high affinity, protecting FVIII from premature and unwanted activation, proteolysis, and clearance (Figure 3) [8].

Besides being the physiologic carrier of FVIII, VWF is an essential mediator of platelet adhesion and aggregation at sites of vascular injury where blood rheology and shear forces are perturbed (Figure 3). To fulfil these roles, VWF interacts with multiple mediators: (1) the VWF-A3 domain binds collagen I and III, which are major components of the extracellular matrix of the cardiovascular system; (2) the VWF-A1 domain interacts with the platelet receptor GPIbα; and (3) the VWF-C4 domain binds to the activated form of platelet αIIbβ3 integrin [9].

The VWF-A3-collagen I/III interaction is not directly dependent on shear forces, but it does require HMWMs to be efficient [10,11]. In physiologic conditions, VWF and collagen are in separate compartments; however, they are in close proximity at sites of injury, facilitating their association and the VWF-platelet interactions. VWF can interact with other components of the extracellular matrix exposed at sites of injury, such as collagen IV, VI, laminin, and fibronectin, and these interactions can contribute to subsequent platelet adhesion. However, VWF binds collagen I/III with higher affinity than to the other components of the ECM, confirming the key role of the VWF/A3-collagen I/III association in hemostasis [12,13]. Importantly, platelets interact with ECM directly, and these interactions synergize with those between VWF and ECM in promoting platelet adhesion.

In contrast, platelet tethering is supported by the VWF-A1 interaction (catch bond) with the ligand-binding domain (LBD) of platelet GPIbα, which is dependent on high shear forces applied to the N- and C-terminal autoinhibitory modules (N- and CAIM), flanking the A1 domain [14]. VWF-GPIbα interaction also requires HMWMs and contributes to platelet activation [15]. VWF also binds heparin-binding growth factors via the same region, modulating extrahemostatic functions of VWF, such as angiogenesis, wound healing [16], and cancer metastasis [17].

The C-terminal region of VWF contains a second platelet interaction site. Among the 6 VWFC domains, the C4 exhibits an Arg-Gly-Asp (RGD) sequence that supports binding to integrin αIIbβ3. This interaction is not directly dependent on shear forces or VWF multimer size. Rather, it is mostly regulated by the activation state and conformation of its ligand. Engagement of the VWF-αIIbβ3 association requires integrin activation and contributes to platelet firm adhesion and aggregation, all necessary steps for thrombus formation under flow conditions [18,19].

Because the size and multimeric profile of VWF are essential for its hemostatic functions, the interactions with and the activity of its protease, a disintegrin and metalloprotease with thrombospondin type 1 repeats, member 13 (ADAMTS-13), are finely regulated. ADAMTS-13 cleaves VWF within the A2 domain at the Tyr^1605^-Met^1606^ bond. The process is shear force-dependent and involves multiple interaction sites that become available on VWF in the A2 and D4-CK domains, ultimately resulting in the precise alignment of the metalloprotease domain of ADAMTS13 with its cleavage site on VWF [20].

### 2.2 Bleeding tendency

The importance of VWF in hemostasis is best illustrated by the occurrence of VWD [7,21]. VWD is a heterogeneous group of bleeding disorders, and the most prevalent of the inherited forms, historically divided into 3 main types. VWD types 1 and 3 (VWD1 and VWD3) are quantitative defects, resulting from reduced/impaired biosynthesis or increased clearance of VWF (VWD1C). VWD type 2 (VWD2) groups the qualitative forms (subtypes A, B, M, and N) associated with abnormal multimeric profiles resulting from defective multimer formation, increased ADAMTS-13–mediated proteolysis, or altered interactions of VWF with its major hemostatic ligands, generally due to genetic variants [7,21].

Most defects that result in bleeding; reduce synthesis; impair multimer formation (VWD2A group I); or prevent VWF interactions with FVIII (VWD2N), collagen, and platelets (VWD2M). Exceptions are genetic variants, clustered in the VWF-A1 domain, that increase VWF interaction with platelet GPIbα and result in VWD2B and genetic variants that enhance VWF sensitivity to ADAMTS-13 proteolysis, resulting in the loss of HMWMs and VWD2A group II.

Although the molecular pathways can be different, bleeding in VWD is mostly mucocutaneous, resulting in easy bruising, extensive bleeding from minor wounds and after surgery, and abnormal nose and mouth bleeding. Moreover, some types of bleeding are characteristic of certain groups of patients. A large proportion (up to 100% depending on the study) of affected women, girls, and people with the potential to menstruate (WGPPM) suffer from abnormal uterine bleeding, gynecological bleeding, and related symptoms [22,23]. GIB, which is often severe, difficult to manage, and can be life-threatening, is more frequent in patients with reduced HMWMs, such as VWD types 2A, 2B, and 3 [[24], [25], [26]].

In case of severe VWF quantitative deficiency and in VWD type 2N, in which genetic variants clustered in the D’D3 region disturb the VWF-FVIII association, a secondary FVIII defect usually manifests as hemophilia A–like phenotypes, such as hemarthrosis and arthropathy, in addition to the VWD phenotypes [27].

Importantly, bleeding in VWD is generally heterogeneous in type, location, and severity and difficult to quantify even with the available and most widely used bleeding assessment tools (BATs; for example, the ISTH-BAT and the pictorial blood assessment charts [PBAC]) [[28], [29], [30]]. Moreover, only in recent years, with the widespread use and development of quality of life (QoL) studies, VWD has been repeatedly associated with physical and mental burden, resulting in low QoL, especially in affected WGPPM [[30], [31], [32]]. In these patients, anemia, iron deficiency, and iron deficiency anemia often develop due to recurring bleeding and contribute to the low QoL, in addition to their effects on blood rheology that negatively impact hemostasis. Available and future treatment options for VWD will be presented later in this article; however, their development and mode of action depend closely on our understanding of VWF functions and dysfunctions.

### 2.3 Thrombosis

Thrombotic manifestations have also been associated with VWF [33,34]. Impaired ADAMTS-13–dependent proteolysis of VWF due to severe ADAMTS-13 deficiency or functional inactivation leads to the accumulation of ultralarge VWF multimers and intravascular thrombosis mainly in small vessels in patients affected by thrombotic thrombocytopenic purpura (TTP) [35]. Moreover, elevated plasma levels of VWF have been associated with thrombotic complications in various settings, including arterial and venous thrombosis, often in association with high FVIII levels, enhancing both platelet deposition and fibrin formation [36,37]. Genetic studies were instrumental in highlighting these associations. On the contrary, several studies have established that VWF deficiency protects from arterial thrombotic events [[38], [39], [40]]. From a molecular perspective, thrombosis is a complex multistep process in which many actors contribute through intricate mechanisms, including coagulation, fibrinolysis, and inflammation, leading to the concept of immunothrombosis, in which VWF has been shown to have multiple roles. Historically, many studies were conducted on the role of VWF in arterial thrombosis, such as myocardial infarction, coronary heart disease, and ischemic stroke (reviewed in the work by Sonneveld et al. [36]). However, it was only more recently that a role for VWF in venous thrombosis was established (reviewed in the work by Michels et al. [37]), and a comprehensive mechanism linking VWF, platelets, neutrophils, and flow was proposed [41].

### 2.4 VWF beyond hemostasis and thrombosis

It has been known for many years now that VWF functions extend far beyond hemostasis and that VWF contributes to many more pathophysiological processes and disorders other than bleeding and thrombosis [42,43]. An unfortunate example is cancer, in which VWF contributes at multiple levels. Indeed, VWF roles in coagulation, inflammation, angiogenesis, vascular permeability, and other pathways link VWF biology and function to cancer-associated thrombosis and dissemination/metastasis (reviewed in the work by Patmore et al. [44]). While these topics will not be discussed in detail in this article, it is worth mentioning that an increasing number of studies in recent years have focused on novel, extrahemostatic, and interconnected functions of VWF and many more will surely address yet unknown VWF-associated mechanisms, interactions, and disorders in the near future. Importantly, deciphering new VWF-dependent functions could help identify VWF as a biomarker and/or therapeutic target in various pathologies and, possibly, improve treatment and care of patients with VWD.

## 3 Genetic Diagnosis and Contributing and Confounding Factors for the Diagnosis of VWD

VWD diagnosis has traditionally relied on clinical bleeding and functional laboratory assays; however, increased understanding of VWF genetics, structure-function relationships, and population genomic variation has made molecular testing an important diagnostic tool [3,4,45,46]. However, interpretation of genetics in VWD is complex, and multiple biological and methodological confounders can inhibit accurate diagnosis and classification.

### 3.1 Genetic basis of VWD

The VWF gene is extremely large and highly polymorphic, with several thousand variants identified [47]. VWD type 1 is inherited in an autosomal dominant pattern, although type 3 and some type 2 subtypes are recessive [48]. Quantitative deficiencies include type 1 (partial deficiency) and type 3 (virtually absent VWF). Type 1 VWD is genetically and phenotypically heterogeneous, with type 1C individuals exhibiting increased clearance [49,50]. However, a substantial proportion of individuals with modestly low VWF (30-50 IU/dL) lack any identifiable pathogenic variants in their VWF gene [51,52].

In contrast, qualitative defects in VWF (VWD type 2) are generally more genetically defined. Type 2A involves impaired multimer assembly or increased ADAMTS13 cleavage, type 2B involves increased affinity for platelet GPIb, type 2M involves defective platelet interactions without multimer loss, and type 2N exhibits reduced FVIII binding [53,54]. Identification of specific variants often clarifies subtype when functional assays are ambiguous and can inform management decisions [55,56].

### 3.2 Utility and limitations of genetic testing

Genetic testing plays a clear role in qualitative VWD and type 3. In type 2B, mutation identification can determine when to avoid desmopressin and instead utilize VWF concentrate [57]. In type 2N, sequencing helps distinguish VWD from hemophilia A and helps clinicians provide accurate genetic counseling [53]. In type 3, the presence of 2 pathogenic variants allows for carrier detection and prenatal assessment [55,58].

By contrast, routine sequencing in type 1 is more controversial. Approximately 40% to 60% of individuals with VWF levels between 30 and 50 IU/dL lack identifiable pathogenic variants using standard approaches [52,59]. Many cases may reflect polygenic determinants or physiological variation instead of monogenic disease [[60], [61], [62]]. Thus, the absence of a variant does not exclude VWD, and results must be interpreted alongside clinical bleeding and biochemical phenotype [46,63].

### 3.3 Modifiers of VWF levels

Multiple biological variables modify VWF levels and influence diagnostic interpretation. The ABO blood group is the strongest known determinant, as individuals with group O have VWF levels ∼25% to 35% lower than non-O individuals [[64], [65], [66]]. As a result, people with blood group O are overrepresented among patients with VWD, comprising ∼77% of all patients with VWD, even though they make up only 30% to 45% of the general population [67].

Environmental factors such as pregnancy, hormonal replacement or oral contraception, stress, inflammation, exercise, aging, or even exposure to cigarette smoke and air pollution have also been shown to contribute to variation in VWF levels [[68], [69], [70]], potentially masking low baseline values [71]. Endocrine disorders such as hypothyroidism may also reduce VWF [72]. Therefore, repeat testing under steady-state conditions and scrutinized preanalytical guidance are recommended.

Genome-wide association studies have identified loci outside VWF that influence circulating VWF concentrations, indicating strong polygenic contributions [[73], [74], [75], [76], [77]]. Thus, many individuals with “low VWF” (30-50 IU/dL) may have normal population variation rather than discrete hereditary disease [62,78,79].

### 3.4 Assay variability and diagnostic confounders

Laboratory assays remain a major source of variability and misclassification. Traditional tests include VWF antigen (VWF:Ag), ristocetin cofactor activity (VWF:RCo), collagen binding, FVIII activity, and multimer analysis [56,80]. VWF:RCo shows substantial analytic variation and poor reproducibility, particularly at low levels [81]. Newer GPIb assays (VWF:GPIbM/GPIbR) improve precision but are not yet globally standardized [82].

Multimer testing allows differentiation between type 1 and type 2A or 2B, but low concentrations can make interpretation difficult [83]. Molecular testing may therefore clarify discrepant or inconclusive phenotypes.

### 3.5 Diagnostic thresholds and overlapping phenotypes

Historically, VWF:Ag <30 IU/dL defines definitive VWD, while a VWF:Ag level of 30 to 50 IU/dL is termed “low VWF,” representing a bleeding risk factor rather than a straightforward disease [62,78,79,84]. Many individuals with low VWF experience mucocutaneous bleeding and may require hemostatic treatment in high-risk settings [50,85]. However, the frequent absence of pathogenic variants in this range has intensified debate regarding whether low VWF represents mild type 1 VWD or population variation influenced by modifiers [51,61]. According to the latest international guidelines, individuals with VWF:Ag levels of 30 to 50 IU/dL are diagnosed with VWD only if an abnormal bleeding phenotype is confirmed [45]. VWF levels increase with age, raising the delicate question of whether or not VWD diagnosis should be re-evaluated in patients with mild quantitative VWF deficiency who show normalization of VWF levels above a certain age [86].

### 3.6 Conclusions on genetics and confounders

The genetic diagnosis of VWD is most definitive in qualitative type 2 subtypes and in type 3 disease, where variant identification correlates strongly with phenotypic abnormalities and provides clinically actionable information [57,58,71]. By contrast, in suspected type 1 VWD and individuals with mildly reduced VWF antigen, so far, genetic testing has limited sensitivity and specificity. Interpretation of results can be confounded by blood type, physiological factors, comorbid medical conditions, stress, anemia, and assay variability. Accurate diagnosis requires integration of data from clinical bleeding history; laboratory assays; and, when available, genetic testing. Continued advances in genomic interpretation and standardization of diagnostic assays may ultimately refine the definition of low VWF and clarify the boundary between normal population variation and true inherited VWD.

## 4 Treatment Choices and Clinical Applications

VWD treatment is guided by type, severity, bleeding pattern, and individual risk factors. Options include DDAVP, plasma-derived (with or without FVIII) or recombinant VWF, antifibrinolytics, and local or systemic hormonal therapy (Table 2) [87]. The challenge of treatment in VWD is mainly due to the heterogeneity of VWD and also due to the complexity of the role of VWF, as an intracellular actor, a stabilizer of FVIII, and a key player in hemostasis, particularly within the microcirculation. This complexity underscores the need for individualized treatment strategies that integrate VWD subtype, bleeding phenotype, clinical context, and patient-specific factors (Table 2).

**Table 2:** Treatment options in VWD.

Treatment	Use cases	Notes, route of administration
Desmopressin (DDAVP)	Type 1 VWD, some 2A/2M	Ineffective in type 3; risk of hyponatremia. SC, IV, intranasal
Antifibrinolytics (eg, TXA)	Mucosal bleeds, adjunct to DDAVP/VWF	Useful for minor surgery or dental procedures. Oral, IV
Plasma-derived VWF/FVIII	All types, especially severe bleeding	Dual-factor products; risk of inhibitors. IV
Plasma-derived high-purity VWF	All types, especially severe bleeding	
Recombinant VWF	Type 3 or DDAVP-unresponsive patients	Pure recombinant VWF not containing FVIII
Efanesoctocog alfa [88]	Case report and clinical trial in VWD3, VWD2N	Fusion protein independent of VWF. IV
Emicizumab (off-label) [89]	Severe/refractory VWD under study in EmiVWD trial (NCT06998524)	Bispecific antibody anti-FIXa and anti-FX. FVIII mimetic. SC
Monoclonal antibody anti-protein S (VGA039)	Under trial in VWD (NCT07115004, NCT05776069)	Rebalancing agent. Increase of thrombin generation in the absence of the FVIII nonhuman primate model of acquired VWD SC.
Bispecific single domain antibody antialbumin and anti-VWF KB-V13A12 [90]	Murine model of VWD1 SC/prophylactic VWD1 (VWD2)	Albumin-bridging of VWF and FcRn-mediated recycling of VWF. SC
Anti-VWF/CK monovalent antibody FcRn-mediated recycling of VWF—HMB-002 [91]	Prophylactic treatment in VWD1 (NCT06754852)	CK domain binding and FcRn-mediated recycling of VWF. SC
BT200/rondoraptivon pegol pegylated RNA aptamer blocking VWF/GPIba interaction [92]	Clinical trials/case reports in VWD2B (NCT07273721)	Decrease of VWF/FVIII clearance, increase in platelet counts (in thrombocytopenic patients). IV, SC

Adapted from Casari et al. [93].

FcRn, neonatal Fc receptor; FIXa, activated coagulation factor IX; FVIII, coagulation factor VIII; FX, coagulation factor X; IV, intravenous; SC, subcutaneous; VWD, von Willebrand disease; VWF, von Willebrand factor.

### 4.1 Desmopressin and adjunctive treatments

Desmopressin (DDAVP) is an established first-line therapy in patients with mild-to-moderate VWD, particularly type 1 and selected type 2 variants [94]. DDAVP promotes the release of endogenous VWF and FVIII from endothelial storage sites, resulting in a transient rise in circulating levels and increased thrombin generation [95]. However, individual responsiveness is variable, and formal test dose is essential to assess its efficacy. In clinical practice, DDAVP is commonly used for minor bleeding episodes and minor surgical or dental procedures in responsive patients. Limitations include tachyphylaxis; the risk of hyponatremia due to its antidiuretic effect; and contraindications in young (age < 2 years) and elderly patients and those with cardiovascular disease or propensity to severe migraines and seizures or those who are heavy smokers. DDAVP is contraindicated in type 2B VWD because of the risk of worsening thrombocytopenia [87]. The recent global shortage of intranasal DDAVP has highlighted the lack of alternatives aside from the subcutaneous route and raises concerns about the potential consequences of future disruptions in the production of this medication.

Antifibrinolytics including tranexamic acid and epsilon-aminocaproic acid are commonly used as adjuncts, especially for mucosal bleeding (eg, epistaxis, oral bleeding, and menorrhagia) [96]. Their safety and oral availability support outpatient, self-managed care. Hormonal therapy—mainly combined oral contraceptives and levonorgestrel intrauterine devices—is widely used to control heavy menstrual bleeding in women with VWD.

### 4.2 VWF replacement therapy

VWF replacement therapy is indicated in patients who are unresponsive or contraindicated for DDAVP, as well as in clinical situations requiring sustained and predictable hemostatic correction. These include prophylaxis, surgery, trauma, and breakthrough bleeds. Plasma-derived VWF concentrates differ in their VWF:FVIII ratios, multimeric composition, and pharmacokinetic (PK) profiles [93]. Recombinant VWF represents an important advance, offering a product with preserved multimeric structure. It is particularly valuable in patients with severe VWD, including type 3 disease, and in those requiring repeated or long-term treatment. High-purity VWF concentrates and recombinant VWF allow endogenous FVIII levels to increase progressively through stabilization by infused VWF, potentially reducing the risk of unwanted excessive FVIII accumulation. This is relevant in patients with major risk factors for thrombosis, such as obesity, major surgery, and cancer. During treatment with VWF-containing concentrates, a minor proportion of patients with type 3 VWD, typically with homozygous partial or complete gene deletions, may develop alloantibodies and some of them may neutralize VWF, rendering replacement therapy ineffective and increasing the risk of anaphylactic reactions. Recently, the development of VWF inhibitor was confirmed to be a rare event with a prevalence of 6% in patients with type 3 VWD [97].

### 4.3 Surgery, invasive procedures, and pregnancy

Treatment choice depends on the VWD subtype, baseline VWF and FVIII levels, previous DDAVP responsiveness, and the bleeding risk associated with the procedure. DDAVP may be sufficient for minor procedures in responsive patients, whereas VWF replacement therapy is generally required for major surgery or in patients with moderate-to-severe disease.

Perioperative management remains a major challenge in VWD. The complexity of VWD in such situations is heightened by 2 key factors. First, the heterogeneity of VWD—encompassing both quantitative and qualitative defects—differentially impacts the functional interaction between VWF and FVIII, leading to considerable variability in FVIII levels among patients. Second, the response to treatment remains difficult to predict whether using desmopressin or VWF-containing concentrates, with or without exogenous FVIII, as these therapies influence FVIII PKs and circulating FVIII levels reflect both endogenous production and the administered factor. A major challenge is the lack of individualized PK data, hindering precise dosing and effective therapy despite population-based PK data being available [98]. This gap is critical during surgery, where achieving sufficient VWF activity without excess FVIII remains difficult. Tailored strategies, guided by preoperative analysis, are needed to optimize outcomes across diverse VWD profiles, especially in high-risk surgical scenarios. Close laboratory monitoring of VWF activity and FVIII levels during surgery or other management needs is essential to guide dosing and duration of therapy. Sustained correction is often required for several days postoperatively, particularly after orthopedic, abdominal, or gynecological surgery. Attention must be paid to excessive FVIII levels, especially in older patients or those with cardiovascular comorbidities, reinforcing the need for individualized dosing strategies. Proactive follow-up and correction of anemia should be in the treatment protocols [99].

Childbirth is a high-risk situation in VWD. Hemostatic support during vaginal or cesarean delivery depends on VWD type and bleeding history. Type 3 and frequently type 2 require VWF concentrates. After physiological increases during pregnancy, VWF and FVIII levels drop rapidly postpartum, raising the risk of delayed bleeding. Extended prophylaxis with VWF concentrates or antifibrinolytics is often needed [28,100].

### 4.4 Prophylaxis and long-term management

Although on-demand therapy remains adequate for many patients, long-term prophylaxis (LTP) is recognized as beneficial in selected individuals with severe VWD and recurrent spontaneous bleeding [87]. This approach is particularly relevant for patients with recurrent epistaxis, joint bleeding, GIB, and menorrhagia. However, LTP remains underutilized in real-world practice. Only a small proportion of patients with VWD receive prophylaxis, with high discontinuation rates related to treatment burden, venous access issues, and patient acceptability. GIB, a major indication for prophylaxis in adults, remains particularly challenging, with limited and inconsistent efficacy of LTP. These constraints disproportionately affect pediatric populations, women with heavy menstrual bleeding, and patients with moderate phenotypes who experience significant disease burden but fall outside traditional prophylaxis criteria.

### 4.5 Emerging therapies and unaddressed clinical needs

Although a century has passed since Erik von Willebrand first described a fatal episode of heavy menstrual bleeding in a 14-year-old girl with type 3 VWD, HMB remains a major clinical challenge, especially among women with milder, often undiagnosed forms such as in type 1 [101]. Despite the significant burden on the quality of life, treatment options are still largely limited to desmopressin and VWF concentrates, contrasting with the progress seen in hemophilia.

Recent case reports and early clinical studies using FVIII mimetics and extended half-life products such as efanesoctocog may offer alternatives or adjuncts to VWF replacement, although they may not fully address platelet-related hemostasis [88,93]. Additionally, nonreplacement therapies targeting downstream coagulation pathways and agents that extend endogenous VWF half-life are under development and show promising potential [90,91].

The heterogeneity of VWD and the emergence of novel therapeutic approaches underscore the need for more appropriate clinical trial endpoints and laboratory vigilance. Traditional measures such as annualized bleeding rate inadequately capture treatment benefit in VWD and should be replaced by bleeding severity, quality of life, menstrual health, pregnancy-related outcomes, and safety endpoints such as thrombosis and inhibitor development [102].

## 5 Concluding Remarks and Future Steps

After 100 years of progress in VWD, still some fundamental challenges remain. Novel functions and interactions of VWF with previously unknown partners highlight the complexity and multifactorial nature of hemostasis, which is directly and indirectly interconnected with many other pathophysiological pathways, some of which still need to be investigated. Despite incontestable progress in genetic testing, advances are expected to increase sensitivity and specificity, especially for type 1 VWD. The early recognition of VWD based on symptoms and laboratory testing is critical and can be improved, especially in WGPPM. Improved awareness and strengthened multidisciplinary medical education are required as nonhematology experts in multiple disciplines are often the first to encounter patients with VWD, including emergency physicians, gynecologists, and gastroenterologists. Thus, the multidisciplinary focus needs our attention. Innovative laboratory testing considering blood flow, impact of blood cells, and interactive glycan- and protein-protein interactions is awaited for both diagnostic and treatment monitoring purposes and to assess the safety and efficacy of novel treatment options.
